# 2D semiconductor nonlinear plasmonic modulators

**DOI:** 10.1038/s41467-019-11186-w

**Published:** 2019-07-22

**Authors:** Matthew Klein, Bekele H. Badada, Rolf Binder, Adam Alfrey, Max McKie, Michael R. Koehler, David G. Mandrus, Takashi Taniguchi, Kenji Watanabe, Brian J. LeRoy, John R. Schaibley

**Affiliations:** 1 Department of Physics, University of Arizona, Tucson, AZ 85721 USA; 2 College of Optical Sciences, University of Arizona, Tucson, AZ 85721 USA; 3 Department of Materials Science and Engineering, University of Tennessee, Knoxville, TN 37996 USA; 4 Materials Science and Technology Division, Oak Ridge National Laboratory, Oak Ridge, TN 37831 USA; 5 Department of Physics and Astronomy, University of Tennessee, Knoxville, TN 37996 USA; 6 National Institute for Materials Science, Tsukuba, Ibaraki 305- 0044 Japan

**Keywords:** Nonlinear optics, Nanoscale materials, Nanophotonics and plasmonics

## Abstract

A plasmonic modulator is a device that controls the amplitude or phase of propagating plasmons. In a pure plasmonic modulator, the presence or absence of a plasmonic pump wave controls the amplitude of a plasmonic probe wave through a channel. This control has to be mediated by an interaction between disparate plasmonic waves, typically requiring the integration of a nonlinear material. In this work, we demonstrate a 2D semiconductor nonlinear plasmonic modulator based on a WSe_2_ monolayer integrated on top of a lithographically defined metallic waveguide. We utilize the strong interaction between the surface plasmon polaritons (SPPs) and excitons in the WSe_2_ to give a 73 % change in transmission through the device. We demonstrate control of the propagating SPPs using both optical and SPP pumps, realizing a 2D semiconductor nonlinear plasmonic modulator, with an ultrafast response time of 290 fs.

## Abstract (web summary)

Plasmonic modulators have many possible applications in optical-frequency devices. Here the authors report a 2D semiconductor nonlinear plasmonic modulator enabled through strong interaction between the surface plasmon polaritons and excitons in a monolayer semiconductor integrated on top of a metallic waveguide.

## Introduction

Plasmonic modulators have been highly sought after for approaches to optical frequency information processing devices^1–5^. Optical frequency plasmonic devices offer potential advantages over electronic devices due to the high carrier frequency of optical waves, as well as the potential to use ultrafast solid-state nonlinearities for sub-picosecond switching times. Furthermore, by using plasmonic structures, optical frequency waves can be confined to sub-free-space wavelength waveguides allowing for miniaturization of on-chip optical devices^1^. Early plasmonic modulators demonstrated modulation using quantum dots^3^, photochromic molecules^6^ as integrated nonlinear materials, but were limited by slow (>40 ns and 10 s) switching times^3,6^. Ultrafast (~200 fs) plasmonic modulation was demonstrated with a modulation depth of 7.5% (~0.3 dB) by direct optical pumping of bare metallic plasmonic waveguides^5^, but required the use of high ~90 nJ pump pulse energy. The current state-of-the-art plasmonic modulators based on traditional bulk nonlinear materials and nanoplasmonic resonator/interferometer structures typically can achieve modulation depths on order of 1–10 dB μm^−1^ with response times >2 ps and require ~3–20 pJ of pulse energy to operate^7–9^. Recently, graphene-based all-optical^10^ and plasmonic^11,12^ modulators have been studied, and compared favorably to previous modulators in terms of switching speed and energy^11^. In 2018, a graphene-based plasmonic modulator achieved 0.2 dB μm^−1^ modulation depth with a switching energy of 155 fJ and a response time of 2.2 ps using a deep sub-wavelength plasmonic waveguide^12^. This progress motivates the investigation of other atomically thin materials in plasmonic modulator structures, that have the potential to achieve faster response times, lower switching energies and larger modulation depths.

In free space optical measurements, monolayer WSe_2_ and other semiconducting TMDs are known to exhibit large light-matter interactions and large third-order nonlinear optical susceptibilities near their exciton resonance^13–19^. Recently, there has been significant interest in using monolayer TMDs for plasmonic applications including the demonstration of SPPs coupling to dark excitons in monolayer WSe_2_^20,21^, increasing the nonlinear response using localized plasmonic effects^22–24^, and enhancement of single quantum emitter emission rates^25,26^

**Fig. 1:**
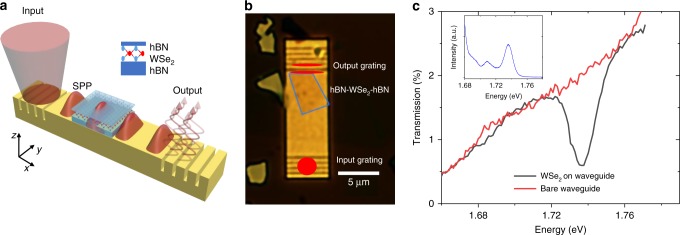
Plasmonic modulator device and linear response. **a** Depiction of the 2D material plasmonic device. SPPs are launched at the input of the device by focusing a free space laser onto an input coupler. The SPPs propagate through the waveguide where they can interact with excitons in the active WSe_2_ layer, encapsulated in hBN. The SPPs are coupled back to free space photons by an output grating coupler. The upper inset depicts the hBN-WSe_2_-hBN heterostructure on top of the waveguide. The lower inset shows the axes used for the theory and simulations. **b** Optical image of the main device used in the experiments. The red dot shows where the grating was illuminated with the input laser, and the red slits show the typical far-field output profile. The blue box shows the location of the active hBN-WSe_2_-hBN heterostructure. **c** Transmission data of the hBN-WSe_2_-hBN plasmonic device (black). The normalized transmission of the bare waveguide is shown (red). The inset shows the far-field PL spectrum of the device when excited with a 532 nm (2.33 eV) laser

In this work, we investigate the 2D semiconductor-plasmonic structure as depicted in Fig. 1a in order to understand the fundamental exciton-SPP interactions and to demonstrate their promise for active plasmonic devices. Our results rely on the atomically thin nature of the TMD and the surface confined SPP mode to realize an attractive geometry where the active layer is near the maximum amplitude of the SPP mode. Furthermore, we develop a novel, self-consistent theory of exciton-SPP (E-SPP) coupling that is unique to the 2D layer geometry and includes a complete E-SPP dispersion relation for arbitrary distances between the metal surface and the TMD layer. We show that our E-SPP model is highly predictive for both the linear (transmission) and nonlinear (differential transmission) response. We take advantage of the fast nonlinear optical response of 2D semiconductor excitons to realize an ultra-low switching energy plasmonic modulator with a modulation depth of at least 4.1% in continuous wave (CW) measurements, limited by the pump power used in the measurement. Our time-domain measurements reveal a fast (slow) component of the nonlinear response with a response time of 290 fs (13.7 ps).

## Results

### Fabrication of E-SPP device and linear response

Monolayer WSe_2_ was integrated on top of the metallic waveguide structures to serve as a nonlinear active layer. Monolayer WSe_2_ was isolated through mechanical exfoliation from high quality bulk crystals. The WSe_2_ thickness was confirmed by photoluminescence. In order to electrically isolate the WSe_2_ from the metallic waveguide (to avoid quenching of excitons), it was encapsulated with hBN. The hBN-WSe_2_-hBN heterostructures were fabricated and transferred onto the waveguides using a polymer based dry transfer technique (polycarbonate film on polydimethylsiloxane, PDMS, stamp)^27^. The transfer was performed under a microscope based probe station to allow for alignment of the 2D heterostructure and waveguide. All of the CW measurements in the main text were performed on the same sample with 5 nm top and 2 nm bottom hBN thickness. Time-domain measurements were performed on another sample with 9 nm and 3 nm hBN thickness. An optical microscope image of a hybrid 2D material plasmonic structure is shown in Fig. 1b.

The hybrid hBN-WSe_2_-hBN/plasmonic structures were measured at 4.5 K (CW measurements) and 11 K (time-domain measurements) in a closed-cycle optical cryostat to reduce thermal broadening effects. The transmission spectra and CW nonlinear measurements were measured using two tunable Ti:sapphire continuous wave lasers (M Squared SolsTiS). The laser was focused to a diffraction limited spot on the input grating coupler. Light scattered from the output grating coupler was isolated using a spatial filter and measured with a silicon photodiode. In the linear transmission measurements, the probe laser was modulated for lock-in detection. In the nonlinear spectroscopy measurements, pump and probe beams were amplitude modulated at different frequencies near 500 kHz to allow for lock-in detection at the modulation difference frequency. The time-domain pump-probe measurements were performed with a tunable mode-locked Ti:sapphire laser with a repetition rate of 76 MHz and pulse width of ~120 fs.

The SPP transmission spectrum is shown in Fig. 1c for 60 µW input power. The black data show the transmission spectrum for the hybrid hBN-WSe_2_-hBN/plasmonic structure (with a ~4-µm-long WSe_2_ layer), and the red data show a reference bare waveguide. At the exciton resonance (1.737 eV, 713.6 nm), the transmission is reduced by ~73% due to the presence of the WSe_2_ layer, indicating a large interaction between SPPs and WSe_2_ excitons. By comparing these transmission data to the photoluminescence spectrum (Fig. 1c inset), we can identify the dip in the SPP transmission as originating from the WSe_2_ neutral exciton (X^0^)^14^. We note that the center energy of the PL and SPP absorption response are aligned to within 1 meV, consistent with previous optical measurements on monolayer WSe_2_^14^.

### Theory of E-SPP

In order to understand the coupling between SPPs and excitons, we use an extension of the well-known SPP dispersion (*k*_*x*_(*ω*)) that relates the wave vector component *k*_*x*_, where the axis *x* is shown in Fig. 1a, of a mode propagating along the surface to its energy ($\hbar \omega$) where *ω* is the SPP’s angular frequency. Our approach complements other theories such as coupled oscillator (plexciton)^28,29^, scattering^30^, and gain-assisted SPP theories^4,31,32^. It is formulated for arbitrary distances between the metal surface and the TMD layer, and reduces to an expression calculated previously^33,34^ in the limit of vanishing distance. Using the dielectric function of the metal *ε*_*m*_(*ω*) and the optical susceptibility *χ*(*ω*) of the TMD layer, we obtain their coupling directly by solving the dispersion relation, which is free of any fitting parameters. We use subscripts 1, 2, and 3 to denote the region above the TMD layer, between the metal surface and the TMD layer, and inside the metal, respectively (Supplementary Fig. 1).

**Fig. 2:**
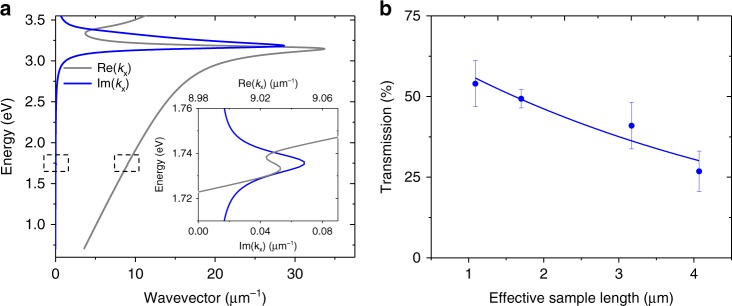
Linear response of exciton surface plasmon polaritons. **a** Calculated real (gray) and imaginary (blue) parts of the dispersion relation for exciton surface plasmon polaritons, E-SPPs. The inset shows the dispersion near the exciton resonance (1.737 eV), whose location is depicted by dashed boxes on the main figure. **b** Measured transmission at the exciton resonance as a function of the effective WSe_2_ sample length on the waveguide

The dispersion relation of the coupled exciton surface plasmon polariton is obtained in a way that is analogous to deriving that of an SPP, i.e., looking for non-trivial solutions of Maxwell’s equations that satisfy the continuity relations at the surface and decay away from it. The difference is the presence of the TMD layer, which requires additional continuity relations to be fulfilled, and there is no exponential decay in the region between the metal surface and the TMD layer. The coupling strength between the surface plasmon and the exciton in the TMD layer is governed by the factor $e^{ - {\mathrm{Im}} k_{2z}z_\ell }$ where *k*_2*z*_ is the wave vector component normal to the surface in region 2, and $z_\ell$. As expected, the coupling is strong only if the layer is within the region of the evanescent surface mode. In the limit $z_\ell \to 0$, we obtain *k*_2*z*_(*ε*_*m*_*k*_2*z*_ − *k*_3*z*_) + *gk*_3*z*_ = 0 where $k_{iz} = \pm \sqrt {\omega ^2\varepsilon _i/c^2 - k_x^2}$ is the wave vector component perpendicular to the surface, the dielectric functions are *ε*_1_ = *ε*_2_ = 1 for the vacuum regions and *ε*_3_ = *ε*_*m*_, and $g = 4\pi i\left( {\omega ^2/c^2 - k_x^2} \right)\chi (\omega )$ provides the coupling, in agreement with previous works^33,34^. The more general form where the distance is an arbitrary input parameter is given (Supplementary Note 1). We use a Drude model for the metal and a Lorentz model for the TMD exciton. This results in an E-SPP resonance at the exciton energy (1.737 eV) with the real and imaginary parts of *k*_*x*_(*ω*) shown in (gray and blue curves, Fig. 2a). The E-SPP group velocity is plotted (Supplementary Fig. 2). The peak value for ${\mathrm{Im k}}_{\mathrm{{x}}}$ of 0.07 µm^−1^ corresponds to an absorption length of 7.1 µm. Figure 2b shows the measured transmission as a function of the effective WSe_2_ sample length for the three different structures we investigated (Supplementary Fig. 3a–f). The effective WSe_2_ sample lengths were estimated from the optical microscope images by calculating an average length over the central 3 µm of the waveguide corresponding the full-width half-max of the SPP spatial mode. An exponential fit to these data yields an effective decay length of 4.8 ± 0.6 µm, which is in good agreement (within a factor of two) of our theoretical model.

### Nonlinear E-SPP interactions

**Fig. 3:**
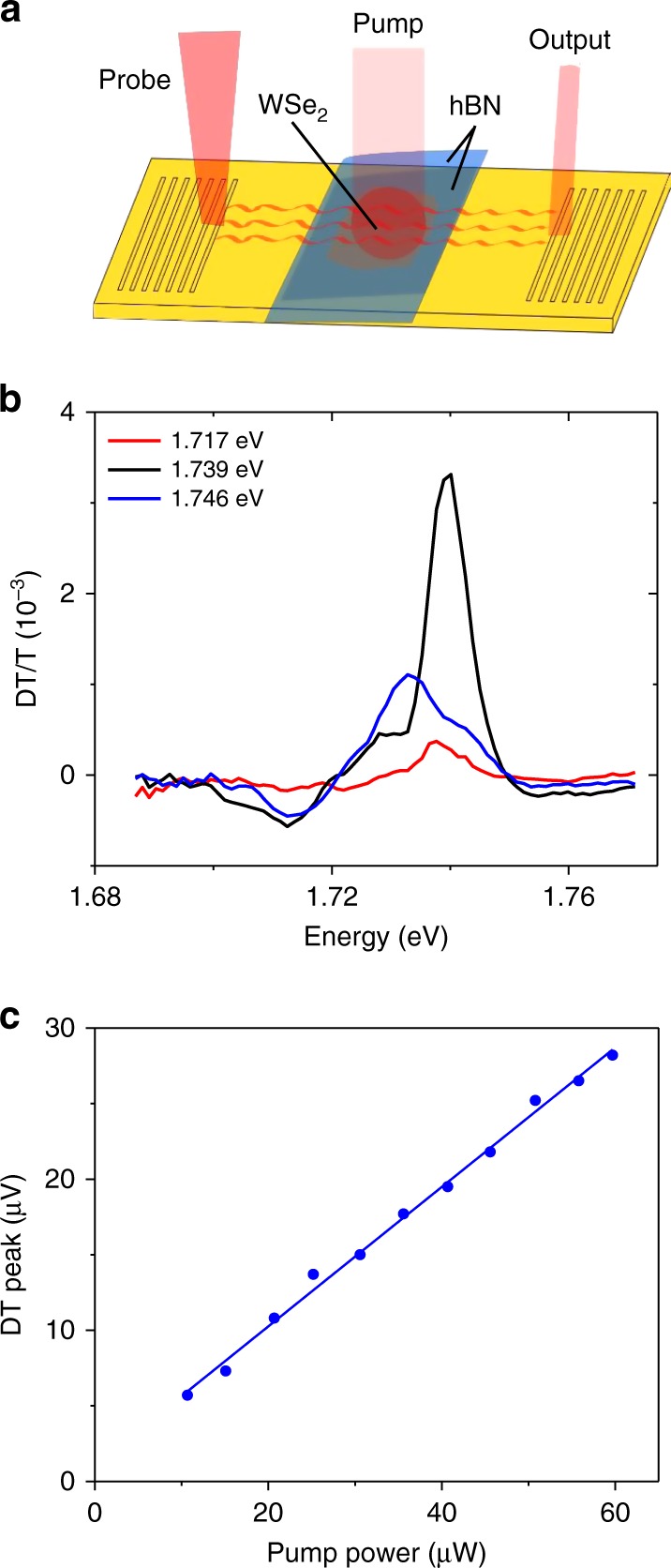
Optical control of SPP propagation. **a** Depiction of the photon-SPP pump-probe measurements. SPPs propagating through the device interact with excitons in the WSe_2_ layer. A free space laser illuminating the WSe_2_ controls the SPP propagation by saturating the exciton absorption. **b** DT/T measurements for three pump photon energies. **c** Pump power dependence of the DT signal near the peak of the exciton response (1.739 eV pump, 1.743 nm probe)

The transmission of the plasmonic device can be controlled by optically pumping the WSe_2_ excitons, partially saturating the absorption. Figure 3a depicts the experimental configuration where SPPs propagating through the plasmonic structure serve as the probe, and a free space laser focused on the hBN-WSe_2_-hBN structure serves as an optical pump. Here, the focused optical pump beam diameter was chosen so that it illuminated nearly the entire WSe_2_ region with an intensity of 8.5 × 10^6^ Wm^−^^2^. Figure 3b shows the DT/T spectrum as a function of probe wavelength, i.e., the pump-induced differential transmission (DT) normalized by the probe transmission (T). DT/T spectra for three different pump energies of 1.717 eV (red), 1.739 eV (black), and 1.746 (blue) are shown. When the pump laser is near resonance with the WSe_2_ X^0^, the DT/T signal is maximized giving a peak value of DT/T = 4.1 × 10^−3^. Figure 3c shows the pump power dependence of the DT signal near the center of the exciton peak (1.739 eV pump, 1.743 nm probe). The DT signal is linear with pump power indicating that the DT response arises from the third-order nonlinear susceptibility.

**Fig. 4:**
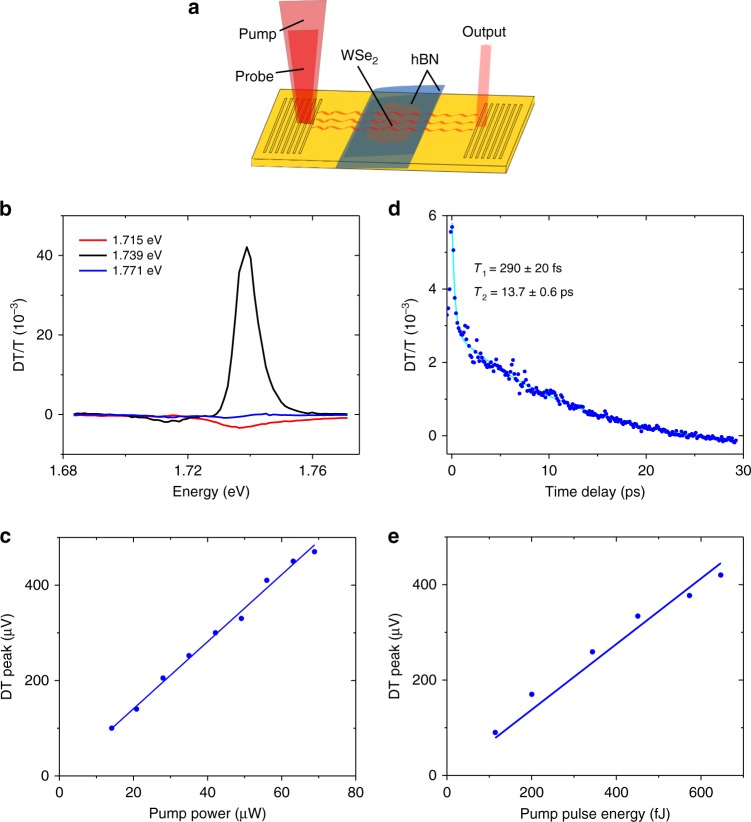
SPP control of SPP propagation. **a** Depiction of the SPP-SPP pump-probe measurements. Pump and probe SPPs are launched at the input grating coupler. SPPs propagating through the device interact with excitons in the WSe_2_ layer. In this plasmonic modulator configuration, the pump-SPP saturates the exciton absorption, resulting in an increase in probe SPP transmission. **b** SPP pump energy dependence of the DT/T spectrum. **c** SPP pump power dependence of the DT signal near the peak of the exciton response (pump 1.739 eV, probe 1.741 eV). **d** Time resolved DT/T response for a similar device measured at 11 K with a photon energy of 1.736 eV. The blue points are the data and the cyan curve is a biexponential fit whose time constants are shown. **e** SPP pump pulse energy dependence of the pulsed DT response measured near zero time delay

In order to demonstrate plasmonic modulation, we performed nonlinear measurements where both pump and probe lasers were coupled into the input grating, launching pump and probe SPPs (depicted in Fig. 4a). Figure 4b shows the CW DT/T spectra for three different pump-SPP energies 1.715 eV (red), 1.739 eV (black), and 1.771 eV (blue). We again observe a strong nonlinear response at the X^0^ resonance, corresponding to a maximum DT/T = 4.1 × 10^−2^. For this case, we see the DT/T amplitude increases by a factor of 10 over the optically pumped signal. The SPP pump power (of the SPP) dependence is also linear (Fig. 4c), consistent with a third-order nonlinear response. From the finite difference time-domain (FDTD) model, we find that the SPP pump intensity is 4.6 × 10^6^ Wm^−2^, ~2 times smaller than the optical pump case.

In order to quantify the response time of the system, we carried out resonant time-domain pump-probe measurements. We used a single (~120 fs) pulsed laser tuned to resonance (1.736 eV, 714 nm) split into pump and probe, and a mechanical delay line to vary the pump-probe temporal separation. The time dependent DT/T response is shown in Fig. 4d. A biexponential is fit to the positive time signal resulting in fast 290 ± 20 fs and slower 13.7 ± 0.6 ps components to the decay time. We note that these decay times are 5–10 times faster than previously reported for monolayer WSe_2_ on SiO_2_^35,36^ which is not surprising due to the coupling to the SPP mode. Figure 4e shows the DT response as a function of pump pulse energy (of the SPP). We note that the required pump pulse energy to achieve a DT/T ≈1% is 650 fJ. We also note that in SPP pump-SPP probe measurements both pump and probe lasers were detected simultaneously, which both contribute to the DT signal. To account for this, the transmission used to calculate DT/T is the sum of both pump and probe beams combined.

To understand this plasmonic modulation effect and to estimate the order-of-magnitude of the third-order nonlinearity, we extended our linear analysis (Fig. 2a) to the third-order nonlinear response with perturbation theory. Since we only observe significant signal near 1.737 eV, we limit our model to in-plane dipoles associated with the X^0^ excitons. This reduces the susceptibility tensor to the single third-order component *χ*^(3)^ (Supplementary Note 1). We assume that the pump-induced change in the susceptibility (Δ*χ*) is proportional to the average pump intensity (*I*_*p*_), i.e. ${\mathrm{\Delta }}\chi (\omega ,I_{\mathrm{p}}) \approx \frac{{4\pi }}{c}I_{\mathrm{p}}\chi ^{(3)}(\omega )$. We can then use the linear dispersion relation with the replacement *χ*(*ω*) → *χ*(*ω*, *I*_p_) = *χ*(*ω*) + Δ*χ*(*ω*, *I*_p_) which yields a pump-induced change in the dispersion, Δ*k*_*x*_, and thus a measure of the pump-induced differential transmission DT/T. We deduce a value for Im*χ*^(3)^ at the peak of the E-SPP resonance by using the experimental value for DT/T and the estimated average intensity. For both, optical pump/SPP probe and SPP pump/SPP probe we find the order of magnitude of Im*χ*^(3)^ to be −10^−20^m^3^V^−2^, in agreement with previously reported all-optical experiments^16,35^. We note this value is a 2D third-order susceptibility. To compare this value to a hypothetical 3D susceptibility, one must divide it by the monolayer thickness.

## Discussion

In this work, we have investigated both the linear and nonlinear response of excitons interacting with propagating SPPs in metallic waveguide structures. We show that the linear absorption of SPPs can be very large, exceeding 73%. The large absorption and nonlinear response might be surprising considering that the out-of-plane spatial extent of an SPP (~400 nm) is much larger than the TMD thickness (0.7 nm). However, our theoretical analysis is consistent with the measurements yielding an absorption coefficient on the order of 0.2 µm^−1^. The key to the large linear absorption is the nanometer-scale proximity of the TMD layer to the metal surface, which allows for the active layer to be located near the maximum of the SPP mode. By performing both optical pump and SPP pump DT measurements, we demonstrate control of SPP propagation with a DT/T response exceeding 4%. The modulation depth per unit length achieved in our modulator (0.04 dB µm^−1^) is within an order of magnitude of other state-of-the-art plasmonic modulators based on monolayer graphene^12^. We note that in both optical and SPP pumped measurements, the maximum pump powers we used were conservatively chosen to avoid sample damage. Since the DT signals are linear in pump intensity up to the highest pump powers used (Figs. 3c and 4c), the reported modulation depths should be taken as lower bounds on the achievable modulation depth.

In principle, the modulation depth could be further enhanced by using longer TMD layers, stacking several TMD layers separated by hBN, or by decreasing the SPP mode size by depositing a high dielectric constant material on top of the structure. The modulation depth can also be increased by utilizing an interferometric or slot waveguide modulator structure^11,12^. Furthermore, our theory predicts that the modulation depth increases with decreasing detuning between the exciton and the SPP resonance. This plasmonic enhancement follows from the equation ${\mathrm{\Delta }}k_x = h_{{\mathrm{enh}}}(\omega ){\mathrm{\Delta }}\chi (\omega )$, where the nonlinear change in the complex-valued propagation vector Δ*k*_*x*_, which governs directly measurable quantities such as DT/T $\left(\propto {\mathrm{Im}} {\mathrm{\Delta}} {\mathrm{k}}_{\mathrm{x}} \right)$ is related to the change in the susceptibility Δ*χ*(*ω*). The plasmonic enhancement factor *h*_enh_(*ω*) is shown (Supplementary Fig. 4). Our model shows that increasing the exciton energy to 2.8 eV (with all other parameters unchanged) would increase Δ*k*_*x*_ by more than 2 orders of magnitude.

To further quantify the performance of our modulator relative to previous works, we consider the response time and minimum energy needed to switch the modulator. Indeed the fast component of our response (290 fs) is comparable to the fastest previously reported plasmonic modulators^5^, but we achieve a similar modulation depth with a ~10^5^ times lower pump pulse energy. Compared to other state-of-the-art plasmonic modulators which typically require ~3–20 pJ of pulse energy to operate, with response times >2 ps^7–9^, our demonstration of a 290-fs response time with 650 fJ pump pulse energy compares favorably. We note that assuming a Gaussian pulse, this response time corresponds to a modulation bandwidth ~1.5 THz. We believe that future 2D semiconductor-plasmonic structures based on our reported nonlinear exciton-SPP plasmonic modulation effect could pave the way towards ultrafast plasmonic amplifiers and transistors with ultra-low switching energies.

## Methods

### Fabrication

The gold waveguide was fabricated on 285 nm SiO_2_/Si substrates by a two-step electron beam lithography process using an electron beam lithography system (100 kV Ellionix) and a spin coated poly(methyl methacrylate), PMMA, resist. In the first step, 200 nm gold was (electron beam) evaporated onto the substrate using 10 nm titanium sticking layer. In the second lithography step, PMMA was respun and the grating pattern was written and developed. We used an Ar^+^ milling process to etch the grating couplers into the waveguide. The waveguides are 5 µm × 13 µm. The grating couplers are composed of five grooves that are 40 nm deep with a width of 110 nm and period of 570 nm. The bare waveguides were characterized using atomic force microscopy (Supplementary Fig. 5a–d) and optical spectroscopy (Fig. 1c). The waveguide and grating coupler designs were optimized using an FDTD model. Simulations of the bare metallic structure show a maximum transmission of ~4% at the exciton resonance (Supplementary Fig. 6). We integrate a hexagonal boron nitride (hBN) encapsulated monolayer transition metal dichalcogenide (TMD) semiconductor, WSe_2_, on top of the waveguide (see Fig. 1a), where the interaction between SPPs and excitons in the WSe_2_ provides the nonlinear response needed for modulation.

## Supplementary information

Supplementary Information

## Data Availability

The data that support the findings of this study are available from the corresponding author upon reasonable request.
