# Supplementary material for: 2D semiconductor nonlinear plasmonic modulators
Source: Nat Commun. 2019 Jul 22;10:3264. doi: 10.1038/s41467-019-11186-w (PMC6646399; doi:10.1038/s41467-019-11186-w)
Supplement: Supplementary file 1 — Supplementary Information [file 41467_2019_11186_MOESM1_ESM.pdf]

Supplementary Information

2D Semiconductor Nonlinear Plasmonic Modulators

Authors: Matthew Klein[1,2], Bekele H. Badada[1], Rolf Binder[1, 2] Adam Alfrey[1], Max McKie[1], Michael R. Koehler[3], David G. Mandrus[3-5], Takashi Taniguchi[6], Kenji Watanabe[6], Brian J. LeRoy[1], and John R. Schaibley[1]

Author Addresses:

[1]Department of Physics, University of Arizona, Tucson, Arizona 85721, USA

[2]College of Optical Sciences, University of Arizona, Tucson, Arizona 85721, USA

[3]Department of Materials Science and Engineering, University of Tennessee, Knoxville, Tennessee, 37996, USA

[4]Materials Science and Technology Division, Oak Ridge National Laboratory, Oak Ridge, Tennessee, 37831, USA

[5]Department of Physics and Astronomy, University of Tennessee, Knoxville, Tennessee, 37996, USA

[6]National Institute for Materials Science, Tsukuba, Ibaraki 305- 0044, Japan

Correspondence to: johnschaibley@email.arizona.edu

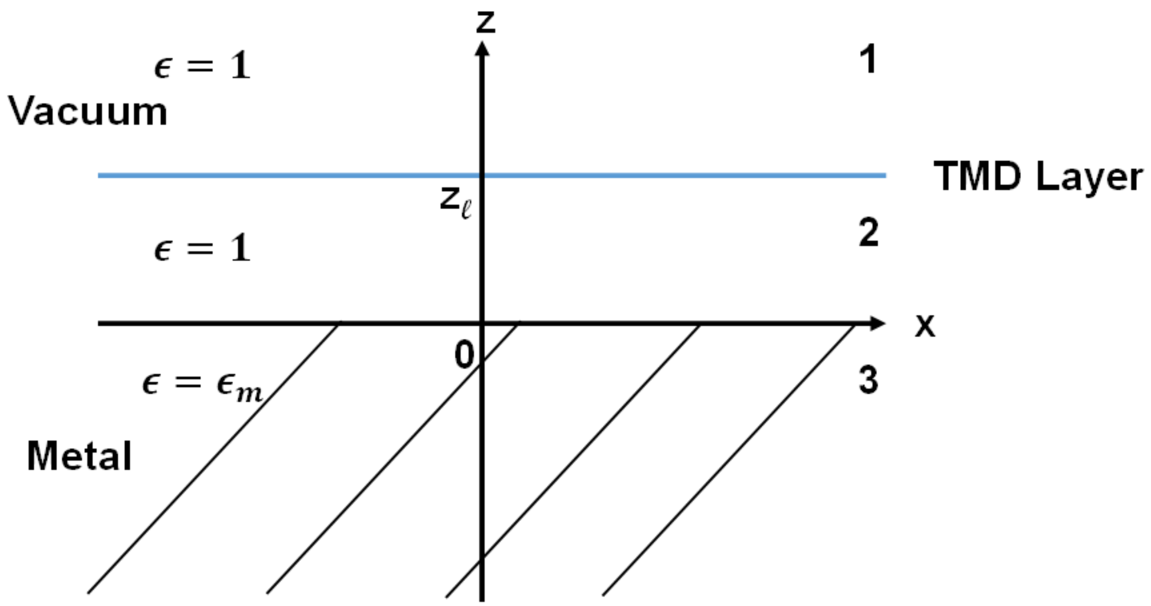

Supplementary Figure 1. Schematic of a TMD layer above the metallic surface. Subscripts 1, 2, 3 denote the region above the layer, between the metal surface and the layer, and inside the metal, respectively.

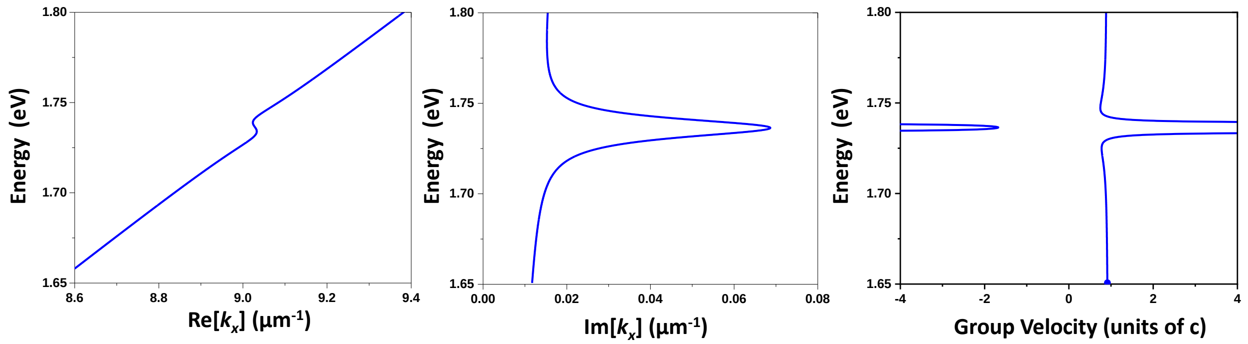

Supplementary Figure 2. Dispersion relation of the E-SPP. **a** E-SPP energy as a function of real part of the wavevector. **b** E-SPP energy as a function of imaginary part of the wavevector. **c** E-SPP energy vs. group velocity.

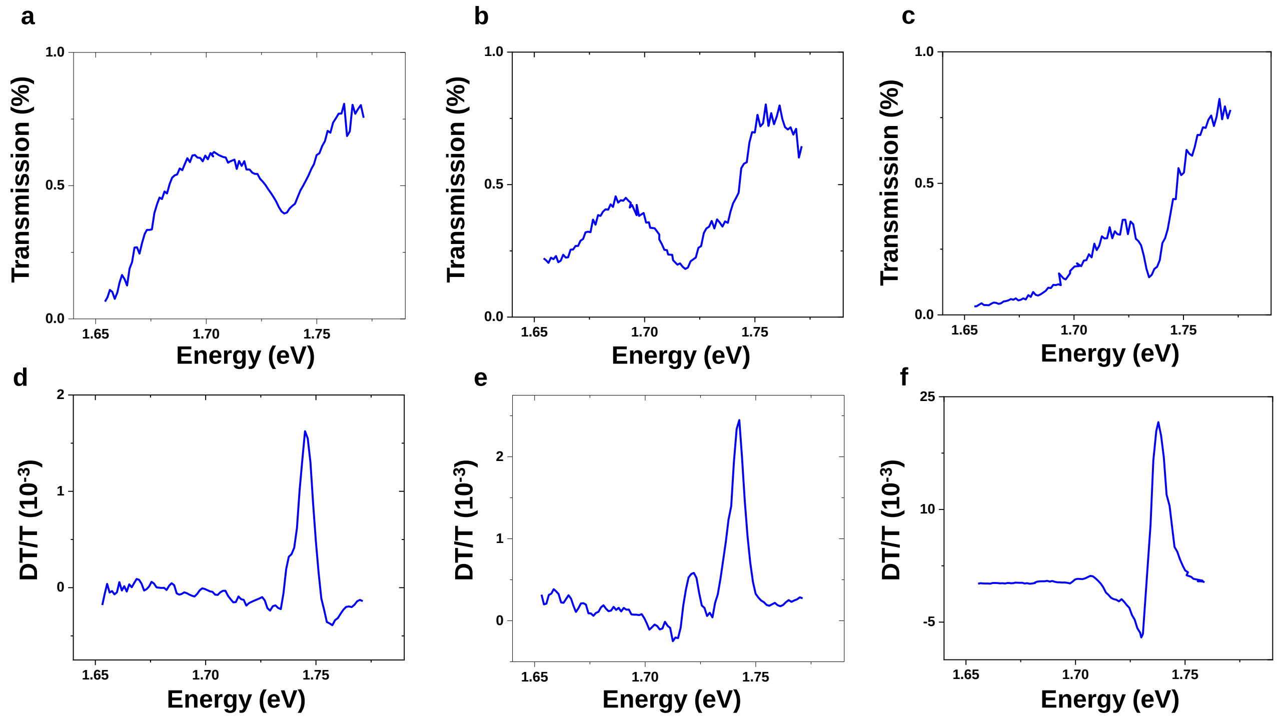

Supplementary Figure 3. Transmission and differential transmission (DT/T) measurements for three additional devices. **a** Transmission spectrum of hBN-WSe_2-hBN plasmonic Device 2 with an effective length of 1.1 µm. **b** Transmission spectrum of hBN-WSe_2-hBN plasmonic Device 3 with an effective length of 1.7 µm. **c** Transmission spectrum of hBN-WSe_2-hBN plasmonic Device 4 with an effective length of 3.1 µm. **d** SPP pump-SPP probe DT/T spectrum (smoothed) for Device 2 at a pump SPP energy of 1.743 eV. **e** SPP pump-SPP probe DT/T spectrum (smoothed) for Device 3 at a pump SPP energy of 1.741 eV. **f** SPP pump-SPP probe DT/T spectrum for Device 4 at a pump SPP energy of 1.74 eV. We attribute the signal near 1.722 eV to the charged exciton (trion). Measurements a-b, d-e (c,f) were performed at 4.5 K (11 K).

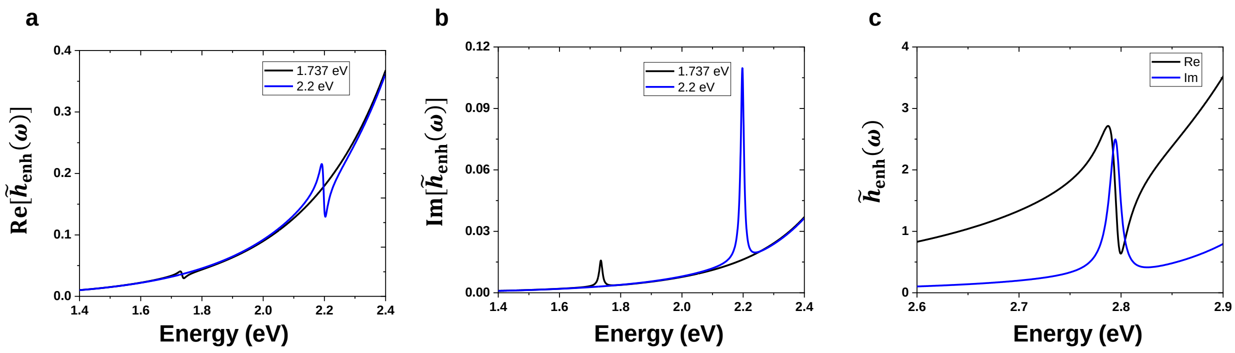

Supplementary Figure 4. Plasmonic enhancement factor $\tilde{h}_{\mathrm{enh}}(\omega)$. (a) Real part for $\hbar\omega_0 = 1.737\,\mathrm{eV}$ (black), $\hbar\omega_0 = 2.2\,\mathrm{eV}$ (blue), (b) same as (a) but imaginary part, (c) real (black) and imaginary (blue) part for $\hbar\omega_0 = 2.8\,\mathrm{eV}$. All other parameters are the same as in Fig. 2a of the main text. The black curve in (a) and (b) corresponds to the exciton frequency in the experiment.

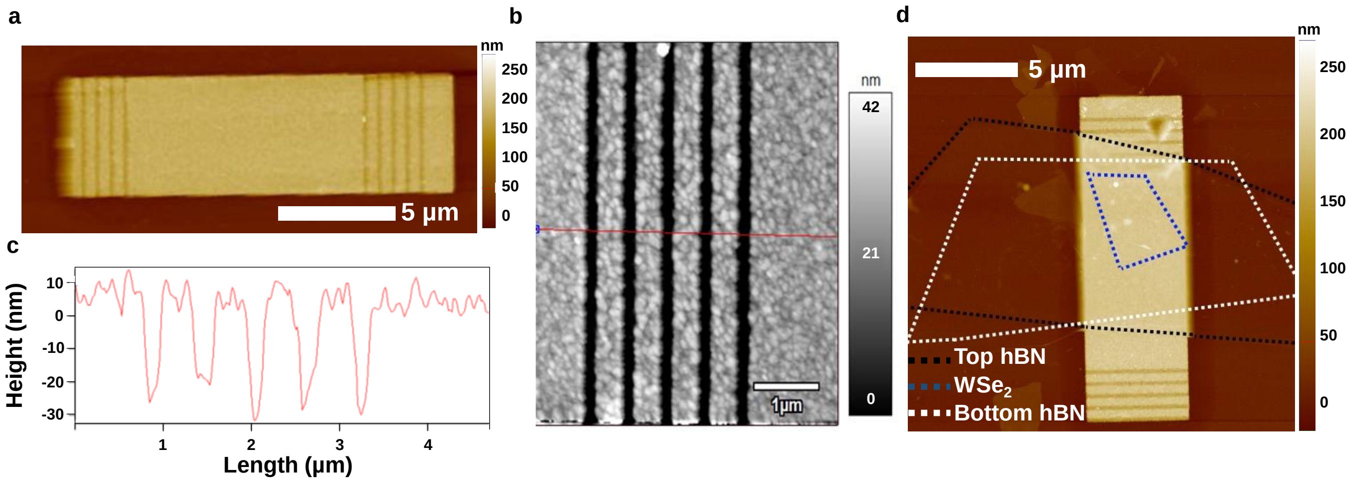

Supplementary Figure 5. Characterization of waveguides. **a** Atomic force microscope image (AFM) of a blank waveguide. The noise on the left side is an artifact of the measurement. **b** Zoom in AFM image of the right grating structure of (a). **c** Line cut along the red line in (b) showing the depth and width of the gratings. **d** AFM of Device 1. The WSe_2 is denoted by the blue dashed line and the hBN are denoted by black and white for the top and bottom pieces respectively.

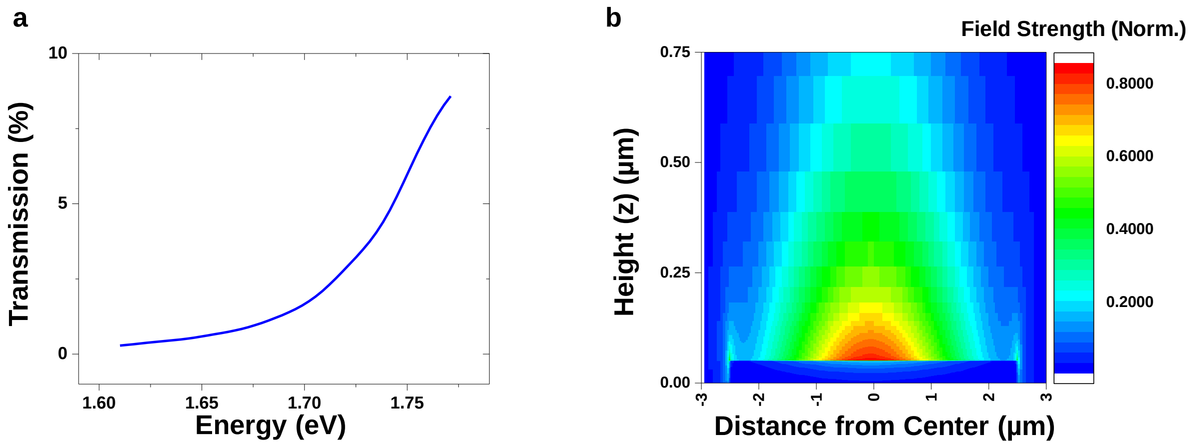

Supplementary Figure 6. Transmission and mode properties of bare waveguide. Finite difference time domain (FDTD) simulation of the bare waveguide including grating couplers. In the simulation, a focused Gaussian beam is incident on the input coupler. **a** Transmission spectrum from the output grating of the waveguide normalized to the input power. **b** Electric field mode of the SPP normalized to the free space field as a function of height above the waveguide and distance from the center of the waveguide (SPP energy is 1.738 eV).

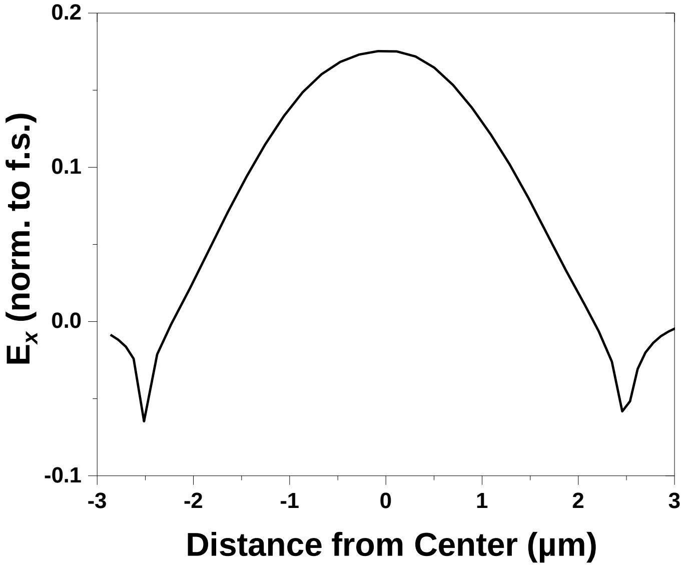

Supplementary Figure 7. Simulated SPP electric field at the location of the TMD layer. FDTD simulation of the x component of the SPP electric field normalized to the focused free space electric field at the location of the TMD Layer.

Supplementary Note 1. Theory of exciton-surface plasmon polariton (E-SPP)

Linear response:

The theory of surface-plasmon polaritons (SPPs) is well established and covered in textbooks. In the following, we proceed along the lines of the SPP theory but include the WSe_2 transition metal dichalcogenide (TMD) layer, which is close to the metal surface and which hosts an excitonic response. We investigate waves that propagate along the surface and are localized in the direction normal to the surface, just as the conventional SPP, we derive a dispersion relation for the coupled exciton-SPP mode, which we call exciton-surface plasmon polariton (E-SPP) modes.

We choose the geometry as shown in (Supplementary Fig. S3), with the metal at $z < 0$ and the TMD layer at $z = z_\ell$, and the system homogeneous and infinitely extended in the x and y directions. We start with Maxwell's propagation equation (in Gauss units)

$$\left\{\nabla^2 - \frac{\varepsilon(z)}{c^2}\frac{\partial^2}{\partial t^2}\right\}\mathbf{E}(x,z,t) - \vec{\nabla}\left(\vec{\nabla}\cdot\mathbf{E}(x,z,t)\right) = \frac{4\pi}{c^2}\mathbf{P}(x,z,t)\,, \tag{1}$$

where $\varepsilon(z)$ is the dielectric constant in each of the three spatial regions indicated in (Supplementary Fig. S3). While the dispersion relation derived in the following is independent of the specific form or the dielectric function in the metal, we note that, in the numerical evaluations we use a simple Drude model,

$$\varepsilon_m(\omega) = \varepsilon_\infty - \frac{\omega_{\mathrm{pl}}^2}{\omega(\omega + 2i\gamma_{\mathrm{pl}})}\,, \tag{2}$$

where ω_{pl} is the plasma frequency, γ_{pl} is a damping constant, and ε_∞, the high frequency dielectric constant. Furthermore, $\mathbf{P}$ is the excitonic polarization of the TMD layer. We utilize a delta-layer approximation,

$$\mathbf{P}(x,z) = \delta(z - z_\ell)\mathbf{P}_{\mathrm{L}}(x)\,, \tag{3}$$

and assume a linear response with respect to the E-field at the position of the layer,

$$\mathbf{P}_{\mathrm{L}}(x,\omega) = \chi(\omega)\mathbf{E}(x,z_\ell,\omega)\,. \tag{4}$$

Below, we extend the formulation to a nonlinear response. Again, the dispersion relation derived in the following is independent of the specific form of the TMD response, but we note that in the numerical evaluations we use the linear excitonic polarization

$$\chi^{(1)}(\omega) = -\frac{c}{2\pi\omega}\frac{\Gamma}{\omega - \omega_0 + i\gamma} \tag{5}$$

where ω_0 is the exciton frequency, Γ the radiative decay rate, and γ the dephasing rate.

We assume p-polarized light in each region. For the two regions $n = 1, 3$, we have $\mathbf{E}_n = (E_{nx}, 0, E_{nz})$ with

$$\mathbf{E}_n(\mathrm{x}, \mathrm{z}) = \mathbf{E}_n e^{i\mathbf{k}_n \cdot \mathbf{r}} \tag{6}$$

and the complex wave vector $\mathbf{k}_n = (k_x, 0, k_{nz})$ corresponding to evanescent waves. Upon neglecting z-excitons in the TMD layer, we can assume the field in region 2 to be of the form

$$\mathrm{E}_{2x}(x,z) = E_{2x}^{+} e^{ik_x x + ik_{2z} z} + E_{2x}^{-} e^{ik_x x - ik_{2z} z} \tag{7}$$

and

$$\mathrm{E}_{2z}(x,z) = E_{2z} e^{ik_x x + ik_{2z} z}\,. \tag{8}$$

Using $\vec{\nabla} \cdot \mathbf{D} = 0$ and standard boundary conditions, we obtain

$$M \begin{pmatrix} E_{1x} \\ E_{3x} \end{pmatrix} = 0 \tag{9}$$

with

$$M = \begin{pmatrix} -k_{3z}(k_{1z} + k_{2z} - g) & u_{-} k_{2z}(\varepsilon_m k_{2z} + k_{3z}) \\ -k_{3z}(k_{1z} - k_{2z} - g) & u_{+} k_{2z}(\varepsilon_m k_{2z} - k_{3z}) \end{pmatrix} \tag{10}$$

where we have defined

$$u_{+} = e^{-i(k_{1z} + k_{2z}) z_\ell} \tag{11}$$

$$u_{-} = e^{-i(k_{1z} - k_{2z}) z_\ell} \tag{12}$$

and

$$g = 4\pi i \left(\frac{\omega^2}{c^2} - k_x^2 \right) \chi(\omega)\,. \tag{13}$$

Furthermore,

$$k_{1z} = \pm \sqrt{\frac{\omega^2}{c^2} - k_x^2}\,, \tag{14}$$

and

$$k_{3z} = \pm\sqrt{\frac{\omega^2}{c^2}\varepsilon_m - k_x^2} , \tag{15}$$

with surface waves requiring $\mathrm{Im}\,k_{1z} > 0$ and $\mathrm{Im}\,k_{3z} < 0$. Finally, $k_{1z} = \pm k_{2z}$, and we choose the plus sign. An implicit dispersion relation is then given by

$$\det(M) = 0 , \tag{16}$$

which onecan solve numerically to obtain the explicit dispersion relation

$$\omega = \omega(k_x) , \tag{17}$$

for real-valued ω and complex k_x. Note that the factor

$$|u_+| = e^{-\mathrm{Im}\,k_{2z}z_\ell} , \tag{18}$$

describes the spatial overlap of the evanescent surface mode with the TMD layer and hence the coupling between the SPP and the exciton. There is only an effective coupling if the layer is within the exponential decay length, given by $1/\,\mathrm{Im}\,k_{2z}$. This length is affected by the excitonic response. In the close-proximity limit, which we define as

$$-\mathrm{Im}\,k_{2z}z_\ell \ll 1 , \tag{19}$$

the dispersion relation simplifies to

$$k_{2z}(\varepsilon_m k_{2z} - k_{3z}) + \mathrm{g}\,k_{3z} = 0 . \tag{20}$$

We see that, without the excitonic response, i.e. with $\mathrm{g} = 0$, this reduces to the standard SPP dispersion $\varepsilon_m k_{2z} = k_{3z}$. We stress that the dispersion relation is a function of the material response functions, i.e. of the dielectric function of the metal $\varepsilon_m(\omega)$ and the TMD response $\chi(\omega)$, and thus free of any fitting parameters for given material response functions.

The numerical results for the linear dispersion presented in the main paper are obtained using the general dispersion relation given above, $\det(M) = 0$. They show that, for our case, the close-proximity approximation is well justified. Finally, we obtain the absorption coefficient for the wave traveling in the x-direction as

$$\alpha = 2\,\mathrm{Im}\,k_x .$$

In our case, the exciton resonance is at $\hbar\omega_0 = 1.737$ eV, and a Lorentzian fit to the linear reflection of the TMD layer on a substrate with refractive index $n_s = 1.77$ yields $\Gamma = 1.645$ meV,

$\gamma = 6.448\,\text{meV}$. Furthermore, we use $\varepsilon_\infty = 6.9$, $\hbar\omega_{pl} = 8.9\,\text{eV}$, and $\hbar\gamma_{pl} = 35\,\text{meV}$. From the numerically obtained dispersion relation, we find the exciton or E-SPP resonance at $\operatorname{Im} k_x = 0.07$ μm^{-1} corresponding to an absorption length of $7.1\ \mu\text{m}$, which is in satisfactory agreement with the experimental estimate of the absorption length. In (Supplementary Fig. S2), we show the complex dispersion (the real and imaginary part of $k_x(\omega)$ in the vicinity of the E-SPP resonance. We also show the group velocity, which exhibits the usual features of a resonance with divergences at the turning points and a region of negative group velocity between them.

Nonlinear response:

In order to obtain a quantitative estimate for the nonlinear response, we proceed as follows. We start with the 3-rd order polarization,

$$P_i^{(3)} = \sum_{jkl} \chi_{ijkl}^{(3)} E_j E_k E_l^*, \tag{21}$$

As above, we neglect z-excitons, so that the induced (nonlinear) excitonic polarization has only an x-component, and correspondingly we consider only the x-components of the E-field at the position of the layer to drive the polarization. The only non-zero polarization component is then

$$P_x^{(3)} = \chi_{xxxx}^{(3)} E_x E_x E_x^*, \tag{22}$$

with

$$P_y^{(3)} = P_z^{(3)} = 0. \tag{23}$$

To further simplify the theory and obtain an estimate for the change of the absorption coefficient, we write the electric field as a sum of pump (superscript p) and probe/signal (superscript s) field

$$E_x = E_x^{\text{p}} + E_x^{\text{s}}. \tag{24}$$

While the field would have to be computed self-consistently in the actual nonlinear E-SPP propagation, we estimate the nonlinearity based on an average value for the intensity $(\sim |E_x^{\text{p}}|^2)$, which in turn we estimate from our experimental data. After linearization in the probe,

$$E_x E_x E_x^* \to 2\,|E_x^{\text{p}}|^2\, E_x^{\text{s}}, \tag{25}$$

we have

$$\chi(\omega) = \chi^{(1)}(\omega) + 2\chi^{(3)}_{xxxx} \, | E^{\mathrm{p}}_x |^2 . \tag{26}$$

We can write this as

$$\chi(\omega) = \chi^{(1)}(\omega) + \Delta\chi(\omega), \tag{27}$$

with

$$\Delta\chi(\omega) \approx \frac{4\pi}{c} I_p \chi^{(3)}(\omega), \tag{28}$$

where I_{p} is the average intensity in the TMD layer. Having the new linear (in the probe field) response $\chi(\omega, I_{\mathrm{p}})$, we can again obtain the dispersion relation, and from that a new $\alpha(I_{\mathrm{p}}) = 2\,\mathrm{Im}\, k_x(I_{\mathrm{p}})$. The nonlinear change in the absorption coefficient is then $\Delta\alpha = \alpha(I_{\mathrm{p}}) - \alpha(I_{\mathrm{p}} = 0)$. The differential transmission is

$$\left.\frac{DT}{T}\right|_{\text{E-SPP}} = e^{-\Delta\alpha\, d} - 1 \approx -\Delta\alpha \; d = -2\,\mathrm{Im}\,\Delta k_x \; d, \tag{29}$$

where d is the E-SPP propagation distance.

It is useful (in order to avoid numerical errors in the subtraction) to determine the relation between Δk_x and $\Delta\chi(\omega)$ from an analytical linearization of the dispersion relation in the close-proximity approximation, which yields

$$\Delta k_x = h_{\mathrm{enh}}(\omega)\Delta\chi(\omega) \tag{30}$$

with the plasmonic enhancement factor for the excitonic nonlinearity

$$h_{\mathrm{enh}}(\omega) = \frac{-4\pi i \left(\dfrac{\omega^2}{c^2} - k_x^2 \right) k_{2z}^2 k_{3z}^2}{k_x \left[g(k_{3z}^2 - k_{2z}^2) + k_{2z}^3 - 8\pi i \chi^{(1)}(\omega) k_{2z}^2 k_{3z}^2 \right]} . \tag{31}$$

We can re-write the relation between Δk_x and $\Delta\chi(\omega)$ in unitless quantities as

$$\Delta k_x d = \tilde{h}_{\mathrm{enh}}(\omega) \frac{1}{d_{\mathrm{layer}}} \Delta\chi(\omega) \tag{32}$$

with $\tilde{h}_{\mathrm{enh}}(\omega) = h_{\mathrm{enh}}(\omega) d_{\mathrm{spp}} d_{\mathrm{layer}}$, where d is again the E-SPP propagation distance and d_{layer} the TMD layer thickness. To see that this factor indeed describes a plasmonic enhancement of the

nonlinearities, we plot $\tilde{h}_{\text{enh}}(\omega)$ in (Supplementary Fig. S4) for a propagation distance of $d = 3\mu\text{m}$ and a layer thickness of $d_{\text{layer}} = 1\text{nm}$. We show three cases that differ only in the value of the exciton energy $\hbar\omega_0$. The figure shows that, for a given value of the excitonic nonlinearity $\Delta\chi(\omega)$, the DT/T signal (which is given by the imaginary part of Δk_x, as well as corresponding nonlinear refractive index changes, given by the real part of of Δk_x) increase with decreasing distance between the exciton and SPP resonance frequencies. Furthermore, the figure shows that $\tilde{h}_{\text{enh}}(\omega)$ is complex-valued, which means that the real part of part of Δk_x is given by both the real and imaginary part of $\Delta\chi(\omega)$ (and similarly for the imaginary part of Δk_x). In other words, the observation of a DT/T signal is based on the real and imaginary part of $\Delta\chi(\omega)$, unlike simple concepts in nonlinear optics, where the differential transmission is given solely by the imaginary part of $\Delta\chi(\omega)$.

To check for consistency, we can compare the value we obtain for $\Delta\chi(\omega)$ from the E-SPP measurements with the one we obtain from conventional differential transmission, DT/T, in normal incidence with the TMD layer on a substrate with refractive index n_{s}. In this configuration, the differential transmission is given by

$$\frac{DT}{T} = -4\pi t_{\text{s}} \frac{\omega}{c} \text{Im}\left\{ \frac{\Delta\chi}{1 - 2\pi i t_{\text{s}} \frac{\omega}{c} \chi^{(1)}} \right\}, \tag{33}$$

where $t_{\text{s}} = \dfrac{2}{n_{\text{s}} + 1}$ is the substrate (amplitude) transmission.

We note that in the limit of an optically thin TMD layer, by which we mean a sufficiently weak linear response, $\left| 2\pi t_{\text{s}} \frac{\omega}{c} \chi^{(1)} \right| \ll 1$, the differential transmission reduces to

$$\left. \frac{DT}{T} \right|_{\text{opt}} \approx -4\pi t_{\text{s}} \frac{\omega}{c} \text{Im}\{\Delta\chi(\omega)\}. \tag{34}$$

While this limit is only approximately fulfilled by our TMD layers, it is still instructive to use it for a rough comparison of the TMD nonlinearity in the E-SPP configuration to that in the all-

optical configuration. Using a measured value for $\left.\frac{DT}{T}\right|_{\rm opt} = 5\times10^{-3}$ at a known (estimated) intensity of $I_p = 12.7\times10^6\,{\rm Wm}^{-2}$ close to the E-SPP resonance, we obtain a value (converted to SI units) for ${\rm Im}\,\chi^{(3)} = -1.3\times10^{-20}\,{\rm m}^3{\rm V}^{-2}$.

To obtain an estimate for $\chi^{(3)}$ from the nonlinear E-SPP propagation we use the measured DT/T together with an estimate of the average pump intensity. In the case of optical pump/SPP probe, we estimate $I_{\rm p} = 8.5\times10^6\,{\rm Wm}^{-2}$, $d = 3\mu m$, and measure $\left.\frac{DT}{T}\right|_{\rm E\text{-}SPP} = 0.0041$ from which we obtain ${\rm Im}\,\Delta k_{\rm x}$. We set, for the purpose of this order-of-magnitude estimate, ${\rm Re}\,\Delta k_x = 0$, which then yields a complex $\chi^{(3)} = -(0.77 + i1.7)\times10^{-20}\,{\rm m}^3{\rm V}^{-2}$. This is consistent with the value estimated from the all-optical measurement given above. In the case of SPP pump/SPP probe, we estimate $I_{\rm p} = 4.5\times10^6\ {\rm Wm}^{-2}$ and measure $\left.\frac{DT}{T}\right|_{\rm E\text{-}SPP} = 0.0412$. This yields $\chi^{(3)} = -(14 + i32)\times10^{-20}\,{\rm m}^3{\rm V}^{-2}$. Given the uncertainties in the experiment and the above-listed simplifying assumptions that enter the theoretical model, this is in satisfactory agreement with the result from the all-optical measurement.

Supplementary Note 2. SPP electric field estimation.

To estimate the intensity of the SPPs, we first calculate the free space, average intensity being coupled into the waveguide. This is done by dividing the power of the beam by the area of a circle with a radius equal to the beam waist, which gives a value of $226\times10^6\,{\rm Wm}^{-2}$. This intensity is then converted into an electric field using $I = \frac{cn\varepsilon_0}{2}|E|^2$, where we take n = 1 (vacuum), yielding a result of $4.12\times10^5\,{\rm Vm}^{-1}$. The free space electric field is then converted into the x component of the SPP field based on the FDTD (Lumerical) simulation (Supplementary Fig. S7) 2 nm above the center of the waveguide (where the TMD layer is in our sample).

We then take the average value of the simulated electric field from y = -1.5 to 1.5 as a scaling factor, equal to 0.147, for converting the free space electric field into the x-component of the SPP electric field of $5.87 \times 10^{4}\,\mathrm{Vm}^{-1}$. This electric field is then converted back into an intensity of $4.57 \times 10^{6}\,\mathrm{Wm}^{-2}$ using the previous equation again taking n = 1.
